# Associations of Objectively-Assessed Smartphone Use with Physical Activity, Sedentary Behavior, Mood, and Sleep Quality in Young Adults: A Cross-Sectional Study

**DOI:** 10.3390/ijerph17103499

**Published:** 2020-05-17

**Authors:** Moisés Grimaldi-Puyana, José María Fernández-Batanero, Curtis Fennell, Borja Sañudo

**Affiliations:** 1 Department of Physical Education and Sports, Faculty of Educational Sciences, Universidad de Sevilla, 41013 Seville, Spain; mgrimaldi@us.es; 2 Department of Didactics and School Organization, University of Sevilla, 41013 Sevilla, Spain; batanero@us.es; 3 Department of Exercise and Nutrition Science, University of Montevallo, Montevallo, AL 35115, USA; cfennell@montevallo.edu

**Keywords:** screen time, smartphone use, sedentary behavior, fitness, sleep patterns

## Abstract

This study assesses the associations of objectively-measured smartphone time with physical activity, sedentary behavior, mood, and sleep patterns among young adults by collecting real-time data of the smartphone screen-state. The sample consisted of 306 college-aged students (mean age ± SD: 20.7 ± 1.4 years; 60% males). Over seven days of time, the following variables were measured in the participants: objectively-measured smartphone use (Your Hour and Screen Time applications), objective and subjective physical activity (GoogleFit and Apple Health applications, and the International Physical Activity Questionnaire (IPAQ), respectively), the number of hours sitting (IPAQ), mood (The Profile of Mood State (POMS)), and sleep (The Pittsburgh Sleep Quality Index (PSQI)). Multiple regressions analyses showed that the number of hours sitting per day, physical activity, and the POMS Global Score significantly predicted smartphone use (adj.R2 = 0.15). Further, participants with low levels of physical activity were more likely to increase the use of smartphones (OR = 2.981). Moreover, mood state (*β* = 0.185; 95% CI = 0.05, 0.32) and sleep quality (*β* = 0.076; 95% CI = −0.06, 0.21) predicted smartphone use, with those reporting poor quality of sleep (PSQI index >5) being more likely to use the smartphone (OR = 2.679). In conclusion, there is an association between objectively-measured smartphone use and physical activity, sedentary behavior, mood, and sleep patterns. Those participants with low levels of physical activity, high levels of sedentary behavior, poor mood state, and poor sleep quality were more likely to spend more time using their smartphones.

## 1. Introduction

There is a global concern suggesting that individuals are not engaging in sufficient amounts of physical activity, participating in high amounts of sedentary activity [1,2], and spending a great amount of time in front of screens (i.e., watching television or using the smartphone) [3]. This is problematic due to the deleterious health effects associated with low amounts of physical activity and high amounts of sedentary activity [4,5], including obesity [6], depression [7], poor sleep [8], and unsatisfactory psychosocial behaviors [7]. Recent studies suggest that screen-based sedentary behavior may have a greater impact on health than overall sedentary time [9]. Due to the swift advances in technology, the smartphone has become an ever-present device that individuals are using for many hours per day [4,5]. It has been established that physical activity and sedentary behavior are independent of one another, because participating in physical activity does not compensate for large amounts of sedentary behavior [10,11]. Therefore, both variables need to be considered independently when assessing their associations with health-related variables.

The use of the smartphone is most prevalent in adolescents and young adults [12,13,14,15,16,17,18]. In the United States, more than 90% of the population above 13 years of age have access to a smartphone, and over 92% of adolescents and 88% of adults use social media platforms [12,13,19]. Research on college-aged individuals has shown that this population uses these devices four to eight hours per day, with the highest users interacting with the device almost non-stop [14,20]. Because interacting with smartphones has become an ever-present activity [15] it is important to understand the health effects of smartphone-based screen time accumulation [16], especially since screen-based sedentary behaviors are directly associated with health harms for young people [17] with evidence for cardiovascular risk factors [18], including higher blood pressure during exercise in youth [21] and low cardiorespiratory fitness [22]. Moreover, screen time ≥2 h per day was reported to be an important risk factor for overweight/obesity in those aged 21–30 years [23,24].

Despite the clear implications for health that the combination of these outcomes (i.e., screen time, mood, sleep, and physical inactivity) may have [25], the evidence is inconclusive [23]. These inconsistencies can be attributed to the different measures of screen time. Most of the evidence comes from self-report questionnaires that rely on the participant’s own perception, which are inferior to objective measures of screen time use due to possible memory bias [26] and underreporting screen time. Studies in the United States that have subjectively measured the association of smartphones with physical activity and sedentary behavior have demonstrated that smartphone use is positively associated with sedentary behavior with no relationship between smartphone use and physical activity and may decrease the intensity of physical activity [27]. There is also evidence that the smartphone is associated with being sedentary and being physically active [28]. Additional inconsistencies include that in the aforementioned studies, subjective measures of physical activity and sedentary behavior were utilized [13,22,29], which may have low accuracy and are likely to have subject bias [30]. Objective monitoring of physical activity levels in large groups of young adults is most commonly accomplished by the use of accelerometers and pedometers because they are cost-effective, accessible, and reliable [31].

Although the relationships between smartphone use and health-related variables in young adults have been tested in the United States [14,20,22], it has not been tested in young adults in Spain. Hence, information about the impact of smartphone use on health-related variables in young adults in Spain is lacking, even when they expend more time on screen-based activities [8,16]. Moreover, all previous studies on this topic are limited to self-reports of smartphone use. To solve these limitations, recent studies have suggested the use of smartphone applications (i.e., apps) that would objectively measure smartphone use directly from the electronic devices, minimizing recall bias [13,16,28]. Therefore, it appears important to examine the effect that objectively measured smartphone time may have on the association between behaviors such as physical activity, sedentary behavior, mood, and sleep among Spanish young adults. Consequently, the aim of the current study is to assess the association between objectively measured smartphone use and these lifestyle determinants (i.e., physical activity, sedentary behavior, mood, and sleep) using built-in sensors equipped in the smartphones to collect real-time data of the screen-state. Because smartphone use has been shown to be associated with sedentary behavior but not with physical activity [14,22], we hypothesize that high smartphone use would be positively associated with sedentary behavior and not associated with physical activity. Moreover, we also hypothesize that high smartphone use would be negatively associated with mood and sleep patterns.

## 2. Materials and Methods

### 2.1. Participants

**Figure 1:**
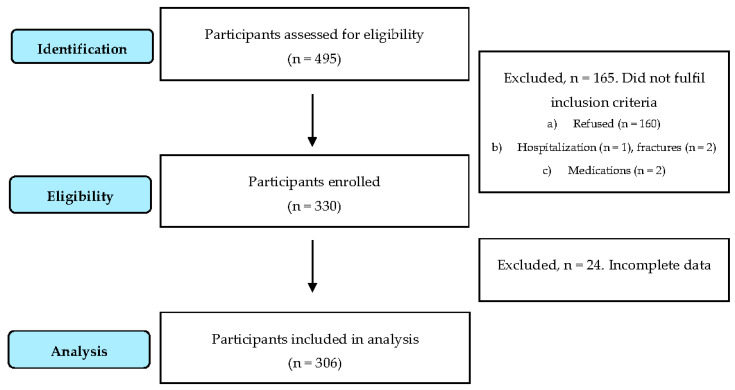
Flow diagram for study participants.

The sample consisted of 306 college students aged 19–25 years (mean age ± SD: 20.7 ± 1.4 years; 60% males) who were selected from different schools in the city of Seville using a stratified cluster sampling method. From the 67 degrees at the University of Seville, a total of nine were surveyed based on their urbanization area. Participants were excluded if (a) they refused to participate in the study; (b) had any health problem that temporarily or permanently prevented participation in physical activity; (c) used any type of medication that could induce changes in the study variables (e.g., opioids or antidepressants). A total of 495 students were initially identified, 160 refused to participate, and five students were excluded for health problems (*n* = 3) or the use of substances (i.e., cannabis) that could induce changes in the study variables. A flow diagram of the study participants is depicted in Figure 1. The study protocol was approved by an ethical committee (0319-N-19).

### 2.2. Procedures

The design of the study included a one week period, during which time spent using the smartphone was automatically registered from their smartphone device, and participants filled out questionnaires on screen use, physical activity, sedentary behavior, mood, and sleep. Researchers visited each school on two separate occasions, separated by one week. During the first visit, the study protocol and its objectives were explained to the participants, and written consent was obtained. Participants were told that their smartphone screen habits, physical activity, sedentary behavior, profiles of mood states, and sleep would be assessed during a one-week period. Then, to objectively measure smartphone use, Android smartphone users were instructed to download the application “Your Hour”, which is a phone addiction tracker and controller (Mind-e-fy Solutions, Madhya Pradesh, India) and iPhone smartphone users were instructed to use the “Screen Time” application (a built-in application on the iPhone) for iOS. The participants were then told and shown how to submit the report after one week. Moreover, they were also instructed on how to collect the number of steps using “GoogleFit”, which is a validated activity tracker that runs in the background of Android devices [32] or the “Apple Health” application for iOS iPhone users, which has been reported to be an accurate step counter in free-living conditions [33]. Separated by one week apart, teachers scheduled sessions with the researchers for those participants interested in participating in the study. Researchers visited each school again and helped each participant to complete the questionnaires and verified that the smartphone use was correctly completed. All of the participants were notified that the data would remain anonymous.

### 2.3. Outcome Measures

In addition to sex and age, information related to education (degree and course), and occupational data (type of employment and the average number of working hours per day) were collected. The main independent variable, objectively measured smartphone use, was measured by how often the student used the smartphone and by the screen state (i.e., amount of screen time) using the applications for the respective smartphone. Objectively measured steps (GoogleFit and Apple Health applications) and subjectively measured physical activity, i.e., frequency, and intensity (using the International Physical Activity Questionnaire (IPAQ)), were measured. Sedentary behavior (i.e., sitting time (using IPAQ)), mood state, and quality of sleep were also assessed.

#### 2.3.1. Smartphone Use and Screen Time

The smartphone screen time accumulated by participants over seven consecutive days was recorded. The smartphone screen-state was objectively captured through the screen-state sensor that recorded the number of times that the screen was turned on or off and the time of the event. Average screen-state time (in minutes) per weekdays and weekends for all users and the number of times that the screen was turned on were used as the outcome measures. To assess screen time, students were asked “How much time per day do you usually spend watching TV, playing video games or using the computer/laptop?”. They were asked to fill in the number of hours and minutes on weekdays and weekends.

#### 2.3.2. Physical Activity

The Spanish version of the IPAQ was used to subjectively measure physical activity and sedentary behavior [34]. This validated questionnaire measures participation in vigorous- and moderate-intensity physical activity and sedentary behavior over the past seven days, and using published values, physical activity data from the questionnaire were transformed into energy expenditure estimates as metabolic equivalent tasks (METs). Outcome measures from the IPAQ were (1) total physical activity expressed as MET-minutes per day and minutes reported in (2) vigorous-intensity, (3) moderate-intensity activity, and (4) in sitting per day. To objectively measure the number of steps, the activity trackers in the participant’s smartphone, described above, were used.

#### 2.3.3. Profile of Mood State

The Profile of Mood State (POMS) [35] is a self-report questionnaire that assesses how the individual has been feeling the past week, including the current day. It consists of 58 items on a 5-point Likert scale from 0 (not at all) to 4 (extremely). The items are grouped in six subscales: Depression (15 items), Fatigue (7 items), Tension (9 items), Confusion (7 items), Anger (12 items), and Vigor (8 items). The total score for each scale was used as an outcome measure.

#### 2.3.4. Pittsburgh Sleep Quality Index

The Pittsburgh Sleep Quality Index (PSQI) [36] is a self-report questionnaire that assesses sleep quality in the past month. It consists of 19 items weighted on a 0–3 interval scale and grouped in seven component scores (subjective sleep quality, sleep latency, sleep duration, use of sleeping medication, daytime dysfunction, sleep duration, habitual sleep efficiency). The outcome measure was the global PSQI score (range, 0–21). PSQI total scores higher than five indicate impaired sleep quality.

#### 2.3.5. Statistical Analysis

Data were reported as means (standard deviations) for continuous variables or percentages for categorical variables. Using multivariate ordered logistic regression, associations between smartphone time with different levels of physical activity, sedentary behavior, mood state, and sleep quality accounting for other covariates (e.g., sex and age) were examined. The validity of the assumptions of the logistic regression model was examined using graphical techniques (i.e., the examination of residual plots). The independent contributions of physical activity, sedentary behavior, mood, and sleep quality on smartphone use were also evaluated by linear regression analysis. Following the clinical guidelines for screen time reported by different institutions, recreational screen time was categorized as <2 and ≥2 h per day [12,37]. Poor sleep quality is defined as PSQI global score >5 [36]. POMS subscales were categorized in quartiles as ‘‘low’’ (quartile 1 representing the lowest score) or ‘‘high’’ (quartile 4, representing the group with the highest scores). Statistical analyses were performed using IBM SPSS Statistics for Windows (Version 23.0., IBM Corp, Armonk, NY, USA).

## 3. Results

The final study sample was composed of 306 young adults (40% females: 20.7 ± 1.6 years; males: 20.8 ± 1.3 years). The data set consisted only of the participant’s data, with all seven days of data recorded. Table 1 presents participant characteristics. Of the 306 participants, 71.3% met the WHO physical activity guidelines of accumulating 150 min/week of moderate physical activity, 75 min/week of vigorous physical activity, or equivalent combination of moderate- and vigorous-intensity physical activity. Of the participants who provided complete sleep data, 30.3% were classified as poor sleepers (PSQI score > 5).

Table 2 illustrates the results of different multiple regressions analyses testing the associations between subjective (IPAQ) and objective (Android and iOS applications) physical activity, sedentary behavior (sitting time), mood, and sleep quality and the time using the objectively-measured smartphone. Sleep quality (standardized coefficients, *β* = 0.076; 95% CI = −0.06, 0.21; *p* > 0.05), and mood state (standardized coefficients, *β* = 0.185; 95% CI = 0.05, 0.32; *p* < 0.001), predicted higher levels of smartphone use. Objectively measured steps (standardized coefficients, *β* = −0.08; 95% CI = −0.20, 0.05; *p* > 0.05) and the POMS Global Score (standardized coefficients, *β* = 0.213; 95% CI = 0.09, 0.34; *p* < 0.001 significantly predicted smartphone use. Additionally, the number of hours sitting per day, subjectively measured physical activity (METs of moderate physical activity) and the POMS Global Score significantly predicted smartphone use. The model explained 15% of the variance in the smartphone use, F_(5, 305)_ = 8.69, *p* < 0.001, Adj.R^2^ = 0.15, with the number of hours sitting per day, *β* = 0.121, *p* < 0.05, total METS in moderate physical activity, *β* = −0.150, *p* < 0.005, and POMS Global Score *β* = 0.176, *p* < 0.001, being a significant predictor of smartphone use.

Table 3 depicts the results of logistic regression models using objectively measured smartphone use as the main outcome variable and subjectively measured physical activity, sitting time, sleep quality, and mood state (POMS subscales) as the predictors. Low levels of subjectively measured physical activity (not meeting the WHO physical activity guidelines) were significantly more likely to increase the use of smartphones (OR = 2.981; 95% CI = 1.325, 7.847). Moreover, those reporting poor quality of sleep (PSQI index >5) were more likely to use the smartphone (OR = 2.679; 95% CI = 1.126, 6.377). Additionally, higher levels of anger (OR = 3.948; 95% CI = 1.450, 10.752) and confusion (OR = 2.756; 95% CI = 1.123, 6.761) were also significantly more likely to enhance the time using their smartphones after controlling for age and sex. Finally, when the regression models were adjusted for the recommendations for screen time (<2 h per day), those participants with greater levels of anxiety were 2.6 times more likely to spend more time using the smartphone (OR = 2.599; 95% CI = 0.639, 10.570), while those with greater levels of depression being 1.7 times more likely to spend more time using the smartphone (OR = 1.686; 95% CI = 0.649, 4.376).

## 4. Discussion

In the present study conducted in a large sample of young adults, we tested whether objectively measured smartphone use was associated with subjective and objective physical activity, sedentary behavior, mood states, and sleep quality. To the best of our knowledge, this is the first study showing that low levels of physical activity, altered mood, and poor sleep quality were independently associated with smartphone use. Additionally, this is the first study conducted that demonstrates that objectively measured smartphone use is independently associated with sedentary behavior. Furthermore, this is the first study in Spain to assess the relationships of smartphone use with physical activity and sedentary behavior. To date, measures of smartphone use were based on self-reported data (questionnaires) and consequently, the findings are not always consistent [38]. Therefore, there is a need to separate the type of measurement of smartphone use across different screen time behaviors (i.e., TV watching or computer use) [39]. The objectively assessed smartphone use patterns reported in the current study may shed some light to understand the health impact of smartphone-based screen time accumulation. Overall, the results of the present demonstrate that more than 40% of our sample spent more than two hours per day using the smartphone. Screen time, especially smartphone use, is a common pastime for young people today, and has led to negative health effects in this population group. These effects of high screen time use include higher risk of being overweight [40]. Moreover, recent studies reported that sedentary behaviors, including an excessive recreational screen time, was independently associated with the metabolic syndrome [41]. Less time for physical activity has also been reported [29]. Recent reports have highlighted that smartphone use is related to sedentary behavior and may have implications for the health of young adults [14,20,22,29]. Therefore, the current data is troublesome because smartphone use is so high and there is increasing evidence showing smartphone use is contributing to negative health behaviors, such as active living [42].

One of the most interesting findings in the current study suggests that those participants with low levels of physical activity are almost three times more likely to increase the use of smartphones. Previous studies in young adults conducted in the United States have reported that screen time was associated with low levels of subjectively measured physical activity [43]. Specifically, related to the present investigation, research that has tested the relationship of smartphone use and physical activity in college-aged individuals have reported no relationship [29]. Researchers have postulated, however, that interaction with the smartphone device may interfere with physical activity, due to its positive association with sedentary behavior (e.g., one may choose to be sedentary when interacting with the device during exercise time) [14]. However, other studies have suggested certain smartphone applications that promote physical activity may increase physical activity [44]. Additional research has demonstrated smartphone use can cause a reduction in the intensity of exercise in both free-living [45] and in a controlled environment [46]. Recently, Tebar et al. [47] suggested that a reduction in the practice of physical activity could be linked to longer times of smartphone use [47], which may have enormous health implications, such as cardiovascular risk [18]. The current investigation has demonstrated a relationship with the smartphone and low physical activity levels, the data also exhibited that, in combination with the time spent sitting, subjectively measured low levels of moderate-intensity physical activity were able to predict more than 12% of the variance of smartphone use. Consequently, this may mean that smartphone use is explained by both sedentary behavior and performing lower levels of moderate-intensity physical activity. Specifically, our data corroborate previous cross-sectional findings that increasing the time spent sitting was positively associated with the smartphone use [29]. Our data also add to the growing literature that young adults expending more time in moderate physical activity reported lower use of the smartphone. Despite this, no such relationships with smartphone use and the number of steps (objectively-measured physical activity) were observed. Therefore, while the smartphone does influence sedentary behavior, it may or may not interfere with physical activity. Further research is warranted on this topic to assess if objective smartphone use influences physical activity.

In the current study, we found that time sitting was positively associated with greater use of the smartphone in college-aged individuals. This is consistent with previous studies that also demonstrated a positive association between smartphone use and sedentary behavior in both college-aged individuals [14,20,29] and those above the college age [13,29]. An important difference with the aforementioned studies is that we used objective measures of smartphone use whereas previous studies have used subjective measures. Because the present findings support results from prior research, it has now been demonstrated that both greater subjective and objective smartphone use is predictive of greater sedentary behavior, both in the United States and Spain [14,48,49]. This is potentially problematic due to excessive sedentary behavior is associated with a higher risk of developing several disease including cardiovascular disease and type 2 diabetes [49]. Therefore, due to its relationship with sedentary behavior, high interaction with the smartphone could contribute to developing cardiovascular disease.

This investigation provides novel information regarding the relationship between smartphone use with mood. Prolonged time spent on screens was reported to be associated with adverse effects on adolescents’ psychosomatic health status [50]. A greater participation in screen-based activities was significantly associated with psychological distress [51]. In the current study, it can be observed that higher levels of anger or confusion were significantly associated with enhanced use of the smartphone. Previous studies reported inconsistent associations between screen time and feelings of nervousness, although they found these associations with the time spent on computers [52]. In the current study, those with higher levels of tension/anxiety (POMS subscale) were two times more likely to spend more time using the smartphone. These findings are in line with Liu et al. [53], who suggested a relationship between mental distress (e.g., depressive and anxiety symptoms) with smartphone use, especially in college students who used smartphones more than four hours per day. In our sample, when the regression models were adjusted for the recommendations for screen time (<2 h per day), those participants with greater levels of anxiety were 2.6 times more likely to spend more time using the smartphone. Finally, some recent studies suggested that both low and high levels of internet use were associated with higher risks for depression [54]. However, contrary to our results suggesting that higher levels of depression were associated with greater time spent with the smartphone, these authors suggested that it seems to be a U-shaped relationship between these outcomes, with both low and high levels of internet use being associated with higher risks for depression. Future studies are warranted to further assess this relationship.

In agreement with previous studies, the relationship between screen-time behaviors and physical activity is complex and not always consistent [55], which could be attributed to the implication of other uses of the smartphone and other related outcomes (e.g., sleep patterns or other sedentary behaviors) [56]. There are several studies suggesting that smartphone use at night is associated with altered sleep patterns [57,58] and sleep disturbances are associated with frequent smartphone use among adolescents [58]. We provide further support to this relationship by demonstrating that those reporting poor quality of sleep (PSQI index >5) were more likely to use the smartphone. As opposed to this evidence, the results of previous studies comparing the associations between screen time and sleep habits are not consistent [43], probably due to the use of subjective (i.e., daily diaries) measures. Despite this, the time spent on screen devices seems to be correlated with difficulty falling asleep and shorter sleep duration [59] which reinforces the idea of a positive association between screen time and disturbed sleep patterns; however, again, we cannot separate the smartphone usage time from the overall screen time (e.g., TV viewing or computer screen time). Leech [52] analyzed the independent contribution of these outcomes on sleep suggesting an association with shorter sleep time. One possible explanation to these interactions are the increments in emotional arousal associated to screen-based activities that might contribute to difficulties falling asleep [53]. Moreover, because screen-based activities are time-consuming, some authors suggest it affects bedtime hour [54].

As practical applications, our findings suggest that young adults’ excessive exposure to smartphones (objectively measured as opposed to subjective assessments of screen time, as previously reported) is associated with lower levels of physical activity, greater sedentary behavior, disturbed mood, and decreased sleep quality. Therefore, it is suggested that a reduction in smartphone use may be important in the prevention of negative health behaviors. Despite these promising results, several limitations must be acknowledged. First, because this was a nonexperimental study, causal inferences cannot be made, and thus, we are not able to determine if reducing the smartphone use can influence the rest of the behaviors (e.g., physical activity, sedentary behavior, disturbed mood, and sleep quality). Furthermore, and despite the study population was a representative sample of students, our results cannot be generalized to different populations or environments. Third, we used self-reported questionnaires to obtain data on physical activity intensity levels, sedentary behavior, mood, and sleep patterns, and while the time spent with the smartphone and physical activity was objectively assessed, there are many other factors that we could not collect (i.e., psychosocial environment or economic status) that may modulate the observed interactions [59]. It is important to note that these behaviors tend to cluster together, and individual relationships may be confounded and may not provide an accurate representation of these important interactions [60]. Finally, in some cases, auto-notifications may not have disconnected, resulting in activation of smartphone screens without the express action of the user that may have been recorded as longer ON-states. Further, being able to see how much individuals are using their smartphone and how much physical activity they are performing may influence their behavior.

## 5. Conclusions

Despite these limitations, it can be concluded that there is an association between objectively measured smartphone use and subjectively measured physical activity, sedentary behavior, mood, and sleep quality. Those participants with high sedentary activity, low levels of physical activity, poor mood state, and poor sleep quality were more likely to spend more time using the smartphones. These results should be taken into consideration by government bodies and health policymakers to understand the impact of excessive use of smartphones on health in order to suggest potential limits to address these issues.

## Figures and Tables

**Table 1:** Characteristics of the participants in the study (n = 306). The data are presented as means (±SD).

Variables	Male (*n* = 184)	Female (*n* = 122)	Total (*n* = 306)
**Age, mean (SD)**	20.8 (1.3)	20.7 (1.6)	20.7 (1.4)
**Sex (%)**	60	40	-
**Professional status, n (%)**			
Work full/part time	112 (62)	68 (38)	180 (59)
Unemployed	72 (57)	54 (43)	126 (41)
**Smartphone use**			
Weekday use (min/day)	266 (94)	238 (105)	254 (99)
Weekend use (min/day)	259 (111)	220 (104)	243 (110)
**Subjectively measured physical activity**			
Walking time (min/week)	264 (171)	240 (165)	253 (168)
Moderate PA (min/week)	192 (153)	114 (136)	157 (151)
Vigorous PA (min/week)	240 (149)	127 (143)	190 (156)
**Objectively measured physical activity**			
Weekday steps (steps/day)	8117 (3439)	7046 (3724)	7623 (3606)
Weekend steps (steps/day)	7848 (3583)	7047 (3938)	7486 (3761)
**Sedentary behavior**			
Sitting time (hours/day)	4.6 (2.4)	5.5 (2.7)	5.1 (2.6)
**Meeting WHO PA guidelines, yes (%) ^c^**	66.8	33.2	71.3
**POMS Global Score ^a^**	22.2 (22.4)	21.0 (22.4)	21.8 (22.4)
Vigor, mean (SD)	16.0 (5.0)	16.3 (4.6)	16.1 (4.8)
Fatigue, mean (SD)	8.6 (6.5)	7.5 (5.7)	8.2 (6.3)
Tension, mean (SD)	9.27 (5.8)	9.6 (5.2)	9.4 (5.6)
Depression, mean (SD)	3.54 (4.9)	3.8 (4.9)	3.7 (4.9)
Anger, mean (SD)	10.6 (6.9)	10.1 (6.6)	10.4 (6.7)
Confusion, mean (SD)	5.92 (4.1)	6.3 (3.9)	6.1 (4.0)
**PSQI Global Score (0–21) ^b^**	3.5 (2.9)	3.9 (3.0)	3.7 (3.0)
**Impaired sleep (PSQI score >5) %**	51	49	30.3

PA, Physical activity. ^a^ POMS, Profile of Mood States. Component scores range from 0 to 3. A higher score indicates greater mood disturbance. ^b^ PSQI, Pittsburgh Sleep Quality Index. Higher scores reflect greater sleep disturbance. ^c^ Meeting World Health Organization physical activity (WHO PA) guidelines: accumulating 150 min/week of moderate physical activity, 75 min/week of vigorous physical activity, or equivalent combination of moderate and vigorous physical activity.

**Table 2:** Individual associations of physical activity, hours of sitting/day, POMS, PSQI, and hours of sitting/day with smartphone-use variables.

Predictor	B	*p*-Value	Adjusted R^2^	*β*	95% Confidence Interval	
					Lower	Upper
PSQI Global Score	16.36	0.265	0.12	0.076	−0.058	0.209
POMS Global Score	4.95	<0.001		0.185	0.052	0.318
Hours sitting/day	22.4	0.155	0.13	0.089	−0.034	0.211
POMS_confusion	36.4	<0.001		0.218	0.097	0.339
N° steps/week	−0.01	0.619	0.13	−0.08	−0.202	0.048
POMS Global Score	5.81	<0.001		0.213	0.091	0.336
Hours sitting/day	4.68	0.049	0.12	0.126	0.003	0.249
METs moderate PA	−0.03	0.012		−0.161	−0.2861	−0.035
Hours sitting/day	4.48	0.048	0.15	0.121	0.005	0.242
METs moderate PA	−0.02	0.003		−0.150	−0.274	−0.027
POMS Global Score	0.69	<0.001		0.176	0.057	0.296

B, non-standardized coefficient; *β*, standardized coefficient; CI, confidence interval; Model, *β* (95%CI): Adjusted for age and gender. POMS, Profile of Mood States; PSQI, Pittsburgh Sleep Quality Index. PA, physical activity; METs: Metabolic Equivalent Tasks.

**Table 3:** Multiple logistic regression of probability of using the mobile smartphone (OR, 95% CI) with physical activity, sleep disturbance, and mood state.

	Predictor	*X^2^*	*p*	Odds Ratio	95% Confidence Interval		
					Lower	Upper	*p*
Smartphone Use	Low Physical Activity	40.9	<0.001	2.981	1.325	7.847	**0.027**
	Sitting time	30.5	<0.001	1.544	0.758	3.163	0.232
	POMS_Vigor	45.2	<0.001	1.115	0.399	3.112	0.835
	POMS_Fatigue	45.0	<0.001	1.644	0.610	4.436	0.326
	POMS_Tension	41.3	<0.001	2.161	0.822	5.681	0.118
	POMS_Depression	46.9	<0.001	2.400	0.948	6.077	0.065
	POMS_Anger	45.5	<0.001	3.948	1.450	10.752	**0.007**
	POMS_Confusion	48.1	<0.001	2.756	1.123	6.761	**0.027**
	Poor sleep quality	39.3	<0.001	2.679	1.126	6.377	**0.026**

OR: Odd ratio adjusted for age and sex; CI, confidence interval. Low physical activity is defined as not meeting WHO physical activity guidelines: accumulating 150 min/week of moderate physical activity or 75 min/week of vigorous physical activity or equivalent combination of moderate and vigorous physical activity. High scores in sitting time are defined as quartile 4, representing the group with the highest times. High scores in the POMS subscales are defined as quartile 4, representing the group with the highest scores. Poor sleep quality is defined as Pittsburgh Sleep Quality Index (PSQI) global score >5. Bold values denote statistical significance at the *p* < 0.05 level.
